# Atypical case of post-partum cardiomyopathy: an overlap syndrome with arrhythmogenic right ventricular cardiomyopathy?

**DOI:** 10.1259/bjrcr.20150182

**Published:** 2015-07-07

**Authors:** G Tse, A Ali, S K Prasad, V Vassiliou, C E Raphael

**Affiliations:** ^1^School of Medicine, Imperial College London, UK; ^2^Cardiovascular Magnetic Resonance Unit, Royal Brompton Hospital, Sydney Street, London, UK

## Abstract

A middle-aged female patient presented with increasing dyspnoea following delivery of her second child. Echocardiography showed left ventricular (LV) dilatation and severe global impairment of systolic function (ejection fraction < 10%) but normal right ventricular (RV) dimensions. Plasma B-type natriuretic peptide level was elevated. Post-partum cardiomyopathy (PPCM) was considered and after initiating appropriate heart failure pharmacotherapy, her symptoms improved significantly. Cardiovascular MR showed RV free wall dyskinesia and aneurysms at the LV apex, RV free wall and RV outflow tract. Genetic analysis showed a C11842T substitution in the titin gene (*TTN*). This is the first case to propose an overlap syndrome of PPCM and arrhythmogenic RV cardiomyopathy.

## Clinical presentation

**Figure 1.**
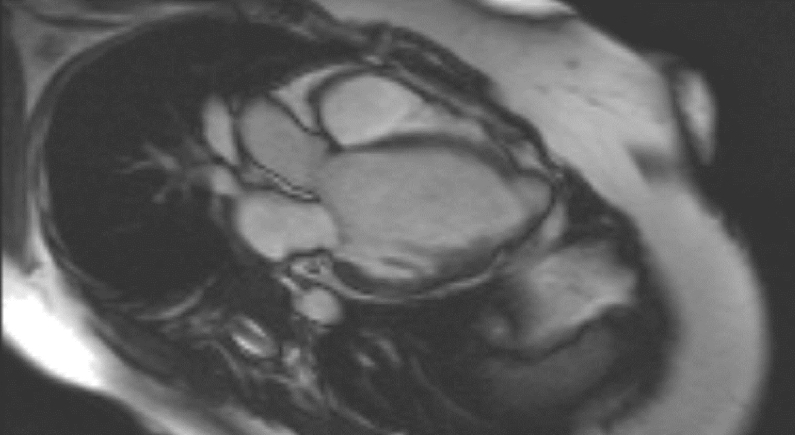
Three-chamber view: aneurysms of the left ventricular apex and the right ventricular free wall.

**Figure 2.**
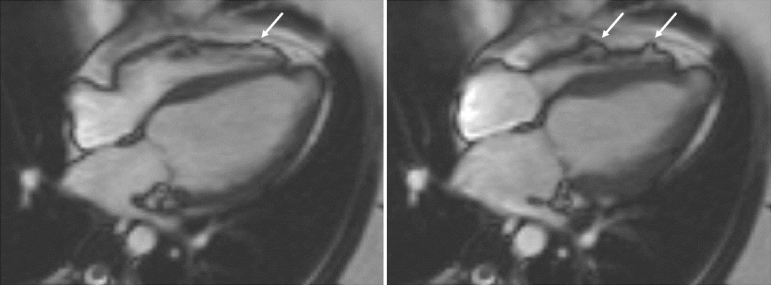
Four-chamber view in diastole (left panel) and systole (right panel). The left ventricle has an apical aneurysm and multiple aneurysms and areas of dyskinesia in the right ventricular free wall are seen (arrowed). This cardiovascular magnetic resonance study had been undertaken 3 months following the initial presentation and the ejection was only mildly impaired, showing a significant improvement when compared with the echocardiogram from 3 months previously.

A middle-aged female patient from a consanguineous family presented with increasing dyspnoea and in New York Heart Association functional class II, 2 weeks after delivery of her second child. Plasma B-type natriuretic peptide level was elevated at 807 pg ml-–1 (normal range: 0–100 pg ml-^–^^1^). Echocardiography showed a dilated left ventricle with severe global impairment of systolic function and an ejection fraction (EF) < 10%, with, however, normal right ventricular (RV) dimensions. Post-partum cardiomyopathy (PPCM) was diagnosed and appropriate heart failure pharmacotherapy was initiated. Subsequently, cardiovascular magnetic resonance (CMR) imaging showed increased left ventricular (LV) end-diastolic and end-systolic volumes and mild global impairment of LV systolic function, confirming an improvement in the systolic function when compared with the initial echocardiogram. RV volumes and function were normal. However, new RV free wall dyskinesia was noted and, additionally, aneurysms at the LV apex (Figure 1; Supplementary Videos A and B), RV free wall [Figure 2, arrow(s); Supplementary Video C] and outflow tract and thinning of the basal septum were observed. Diagnostic coronary angiography revealed unobstructed major epicardial coronary arteries.

## Discussion

**Figure 3.**
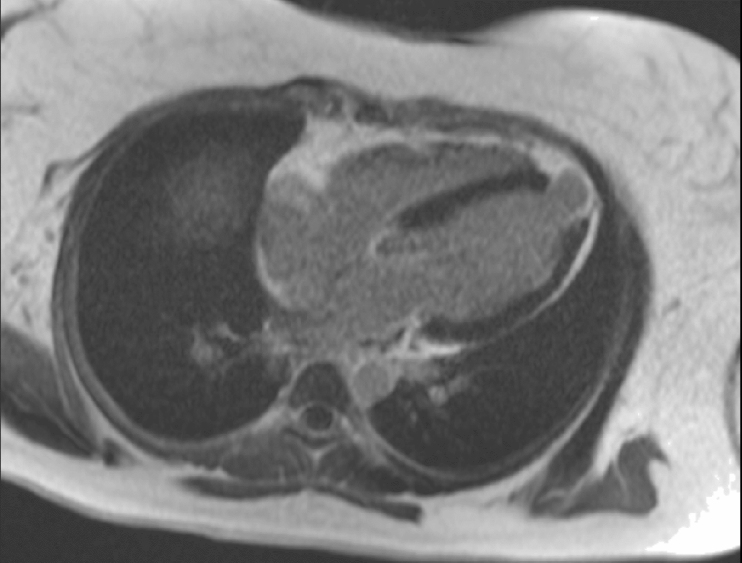
Four-chamber late gadolinium enhancement imaging confirming the left ventricular aneurysm and the apical transmural myocardial fibrosis.

The findings in this female were not typical of an isolated PPCM. While the increased volumes and reduced EF despite normal wall thickness of the left ventricle are consistent with cardiomyopathy, the dyskinesia of the RV free wall together with aneurysms of the RV free wall and outflow tract raise suspicion for a right-sided cardiomyopathic process. These findings are also recognized features of arrhythmogenic right ventricular cardiomyopathy (ARVC). However, in this case, they are without concomitant RV dilatation or impairment required to make a CMR diagnosis by the Revised Taskforce Criteria^1^. As such, this case may represent an overlap syndrome of PPCM and ARVC. The basal septal thinning and transmural infarction of the apex (Figure 3) in the context of normal coronary arteries raised the possibility of arterial recanalization, an embolic episode, coronary spasm or even a process relating to cardiomyopathy itself^2^. The patient had not experienced any symptoms prior to this presentation to suggest a prior acute coronary syndrome event.

In this patient, genetic analysis demonstrated a potential pathogenic mutation of the cardiac myosin-binding protein-C gene *(MYBPC3),* which has previously been shown to be causative for dilated cardiomyopathy (DCM)^3^. In addition, a novel, non-synonymous single nucleotide polymorphism was shown in the titin gene (*TTN*): there was a C11842T substitution, resulting in a single amino acid change from arginine to cysteine at the 3948 position. Titin plays a key role in cardiac sarcomere assembly, force transmission and maintenance of resting tension^4^. *TTN* mutations have previously been implicated in several conditions, including DCM^5^ and ARVC overlap syndromes^6^. No desmosomal gene abnormalities were identified. In our study, we describe a case of RV aneurysms and dyskinesia together with PPCM findings, which is likely to reflect an overlap syndrome.

## Learning points

1. A spectrum of cardiomyopathic processes exists as reflected in overlap syndromes.
2. Cardiovascular MRI is useful for the diagnosis of cardiomyopathy and subsequent disease characterization and future monitoring.
3. *TTN* is a crucial gene that regulates myocardial function.
4. Mutations in *TTN* have been associated with DCM and ARVC.
